# Neutrophils as a Novel Target of Modified Low-Density Lipoproteins and an Accelerator of Cardiovascular Diseases

**DOI:** 10.3390/ijms21218312

**Published:** 2020-11-05

**Authors:** Takashi Obama, Hiroyuki Itabe

**Affiliations:** Division of Biological Chemistry, Department of Pharmaceutical Sciences, Showa University School of Pharmacy, 1-5-8 Hatanodai, Shinagawa-ku, Tokyo 142-8555, Japan; h-itabe@pharm.showa-u.ac.jp

**Keywords:** neutrophil extracellular traps, oxidized low-density lipoprotein, endothelial cells, HL-60 cells, inflammatory response

## Abstract

Neutrophil extracellular traps (NETs) significantly contribute to various pathophysiological conditions, including cardiovascular diseases. NET formation in the vasculature exhibits inflammatory and thrombogenic activities on the endothelium. NETs are induced by various stimulants such as exogenous damage-associated molecular patterns (DAMPs). Oxidatively modified low-density lipoprotein (oxLDL) has been physiologically defined as a subpopulation of LDL that comprises various oxidative modifications in the protein components and oxidized lipids, which could act as DAMPs. oxLDL has been recognized as a crucial initiator and accelerator of atherosclerosis through foam cell formation by macrophages; however, recent studies have demonstrated that oxLDL stimulates neutrophils to induce NET formation and enhance NET-mediated inflammatory responses in vascular endothelial cells, thereby suggesting that oxLDL may be involved in cardiovascular diseases through neutrophil activation. As NETs comprise myeloperoxidase and proteases, they have the potential to mediate oxidative modification of LDL. This review summarizes recent updates on the analysis of NETs, their implications for cardiovascular diseases, and prospects for a possible link between NET formation and oxidative modification of lipoproteins.

## 1. Introduction

Although neutrophil extracellular traps (NETs) were initially considered to be one of the first line responses of the immune system against infected bacteria [1], extensive studies have revealed that NET formation is associated with the initiation and progression of various noninfectious diseases [2,3]. In cardiovascular diseases (CVDs), NETs have been found in vascular lesions such as atherosclerotic plaques and thrombi [4]. Numerous studies have demonstrated that the products released from neutrophils during NET formation directly injure vascular tissues and induce inflammation, indicating that NETs contribute to the progression of vascular diseases.

Increased low-density lipoprotein (LDL) levels in plasma are known to be independent risk factors for atherosclerosis. The molecular structure and characteristics of LDL change dramatically in oxidatively modified LDL (oxLDL). Accumulating studies have revealed that oxLDL is present in both atherosclerotic lesions and circulation [5] and plays a pivotal role in the progression of CVDs by promoting foam cell formation as well as initiating endothelial inflammatory responses [6].

LDL is modified by both enzymatic and non-enzymatic actions. For example, myeloperoxidase (MPO) produces reactive oxygen species (ROS) that cause oxidative modifications of proteins and lipids in lipoprotein particles. Neutrophil elastase (NE) has been reported to degrade protein components in LDL [7], leading to enhanced uptake of modified LDL by macrophages [8]. Neutrophils, in addition to macrophages, are frequently found in vascular lesions; thus, NET formation and the subsequent release of enzymes could presumably potentiate oxLDL production. Furthermore, whether LDL and oxLDL can affect NET formation during CVD development remains unclear. For several years, the biological and pathological effects of neutrophils and lipoproteins on CVDs have been addressed and discussed individually. Insight into the interrelationship between neutrophils and lipoproteins that coexist in the circulation and in vascular lesions under pathological conditions may shed light on a new possibility to understand their roles in vascular diseases.

In this review, we summarize recent updates on the analysis of NETs and their implications for CVDs. A possible link between NET formation and oxidative modification of lipoproteins has also been discussed.

## 2. Induction of NETs

Neutrophils or polymorphonuclear leukocytes (PMNs) have a short lifespan and are highly susceptible to activation. The human promyelocytic leukemia cell line, HL-60, is widely used in the in vitro analysis of NETs. HL-60 cells can be differentiated into neutrophil-like cells using various pharmacological stimulants, such as all-*trans* retinoic acid, dimethyl sulfoxide, and dimethylformamide. HL-60-derived neutrophil-like cells reveal NET formation characterized by CD11b expression, DNA release, oxidative burst, and histone citrullination; however, these responses are more pronounced in PMNs than in HL-60 [9]. Phorbol myristate acetate (PMA), a potent protein kinase C activator, is a well-defined NET-inducer [10]. In addition to PMA, several cytokines and chemokines have been reported to induce NET formation, including TNFα, IL-8 [1], IL-18 [11], CXCL7 [12], complement (C5a) [13], and interferons [14]. Moreover, NET formation is primed by crystals of monosodium urate [15,16] and cholesterol [17]. High concentrations of glucose induce NETosis [18], indicating that NET formation is increased in patients with type 2 diabetes [19] and diabetic retinopathy [20]. Furthermore, nicotine also induces NET formation [21], and PMNs from smokers are more susceptible to PMA-induced NET formation than those from nonsmokers [22].

NET release can be classified into two types: (1) “suicidal NETs”, which proceed in 3–4 h, and induce DNA release concomitantly by rupturing neutrophils to cause cell death (NETosis); and (2) “vital NETs”, which release DNA via vesicles into the extracellular space within 60 min, without causing cell death [2,23]. Notably, the term “NETosis” is applicable only when experimental evidence clearly supports cell death upon NETting [24]. While NET formation is elicited via various signaling pathways involving ROS production [25], the two types of NETs are mediated by different sources of ROS. The Raf-MEK-ERK pathway stimulates NADPH oxidase-mediated ROS production in the process of suicidal NETs [26], whereas mitochondrial ROS is mainly required in vital NETs [21,27]. Chromatin decondensation associated with fragmentation of the nucleus is an important step that releases DNA into the extracellular spaces. MPO and proteases, particularly NE, translocate from azurophilic granules to the nucleus to promote disintegration of the chromatin [28], leading to histone degradation [29]. Gasdermin D is a pore-forming protein that was initially identified as a crucial factor for pyroptotic cell death of macrophages [30,31]. NE-dependent cleavage of gasdermin D activates itself, which in turn plays a pivotal role in suicidal NETs by expanding granules and nucleus, and eventually ruptures the plasma membrane [32,33].

## 3. Detection and Analysis of Protein Citrullination

DNA strands released from neutrophils are decorated by proteins derived from the cytoplasm, azurophil granules, and nuclei. Intracellular ROS production activates peptidylarginine deiminase 4 (PAD4), which catalyzes the posttranslational modification of positively charged arginine residues of histones to neutral citrulline residues. Previous studies have demonstrated the necessity of PAD4 activity for NET formation, whereas most recent studies that have compared the process of NET formation in mouse and human neutrophils, in addition to HL-60-derived neutrophils, have revealed that PAD4 enzymatic activity is crucial for efficient DNA decondensation [34,35].

Because citrullinated histones are considered as biologically and pathophysiologically relevant markers for NET formation, sensitive and accurate detection of citrullinated proteins is crucial for investigation. Although immunological detection is commonly performed for this purpose, notably, antibodies against citrullinated histones vary remarkably [36]. Moreover, besides citrullinated histones, the citrullinated proteins generated during NET development are poorly understood owing to limited availability of antibodies against different citrullinated proteins [24].

**Figure 1:**
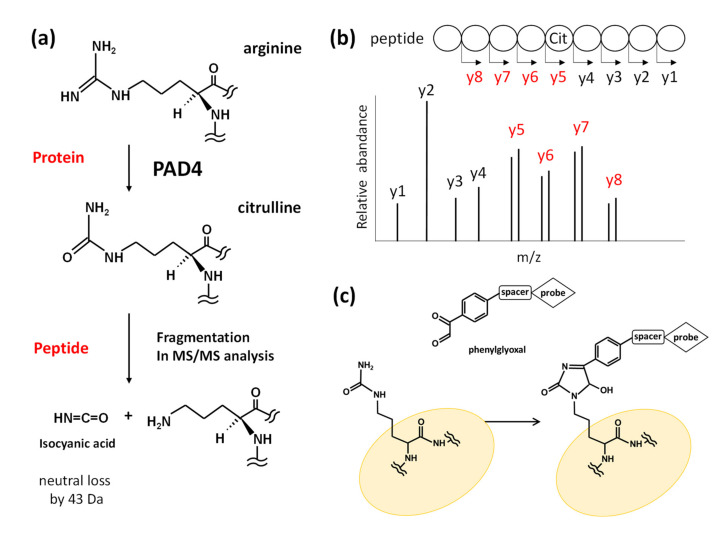
Structures of the citrulline residue and the methods to identify citrullinated proteins using tandem mass spectrometry (MS/MS). (**a**) Deimination of an arginine residue by peptidylarginine deiminase (PAD) 4 produces a citrulline residue. In MS/MS analysis, fragmentation of citrulline releases an isocyanic acid group that causes a 43 Da loss, which can be utilized in a neutral loss scan to detect citrulline residues. (**b**) MS/MS spectra of citrullinated peptides by neutral loss scan can determine the amino acid sequences and positions of citrulline residues. In the secondary MS stage, fragmentation of the peptide occurs at any peptide bond in addition to neutral loss of citrulline residue. The m/z signals correspond to fragments with different numbers of amino acid residues; the fragment ions containing citrulline can be identified as paired spectra with a 43 Da difference. (**c**) Phenylglyoxal probes readily react with citrulline residues to form stable adducts under acidic conditions. These probes contain the structural moiety of rhodamine or biotin to assist specific detection or purification.

Mass spectrometry (MS) effectively analyzes posttranslational modifications without using specific antibodies. Recent developments in MS-based analyses have provided few methods to detect citrullinated proteins. It is difficult to detect specific citrullinated residues in proteins using conventional MS/MS analyses because the technique should be able to detect minute differences in the molecular mass between arginine and citrulline, which is only 0.98 Da; however, citrulline residues release an isocyanidine group via fragmentation reaction during MS/MS analysis. Thus, citrulline residues in a protein can be efficiently identified by detecting a 43 Da difference in the molecular mass, which corresponds to the neutral loss of the isocyanidine group (Figure 1a,b) [37].

An alternative method utilizing a chemical labeling technique has been developed using phenylglyoxal (PG)-based probes that react with citrulline under acidic conditions (Figure 1c) [38]. Presently, two types of PG-based reagents are available: (1) rhodamine-PG, which is used for 2D-PAGE to visualize proteins containing citrulline residues [39], and (2) biotinylated-PG, which is used to enrich citrullinated peptides or proteins. This technique has revealed that numerous proteins comprising citrullinated residues occur in the synovial fluid and synovial tissue of rheumatoid arthritis, where the major citrullinated proteins are serine protease inhibitors such as antiplasmin, antithrombin, tissue plasminogen activator inhibitor, C1 inhibitor, and nicotinamide N-methyltransferase [40].

Most recently, a proteomic approach successfully elucidated a mechanistic link between PAD4-mediated citrullination of proteins and disease conditions [41]. A disintegrin-like and metalloproteinase with thrombospondin type 1 motif 13 (ADAMTS13) is a specific protease for von Willebrand factor (vWF), known to cause thrombotic thrombocytopenic purpura [42]. vWF, a glycoprotein produced by endothelial cells (ECs), forms multimers and binds to collagen and Factor VIII, thus supporting platelet binding to the wound sites. vWF has been elucidated as one of the key factors in the development of NET-mediated endothelial damage and formation of thrombosis. Venous thrombi are characterized by red clots enriched with erythrocytes, because erythrocyte binding to vWF is promoted upon reduction of shear stress [43]. PAD4 released from NET-forming neutrophils citrullinates ADAMTS13; the modified ADAMTS13 loses its proteolytic activity against vWF, leading to the formation of thrombi after vessel injury via accumulation of platelets [41].

## 4. NET-Related Receptors

Membrane receptor-mediated signaling pathways lead to NET formation via ROS production and PAD4 activation [25]. In general, toll-like receptors (TLRs) on the plasma membrane of neutrophils play distinctive roles in NET formation in the presence of bacterial pathogen-derived stimuli, including lipopolysaccharides [25].

The receptor for advanced glycation end products (RAGE) is expressed on human neutrophils [44]. High-mobility group box 1 (HMGB1), a DNA-binding protein secreted from macrophages and monocytes, which acts as a damage-associated molecular pattern (DAMP) to mediate thrombosis [45], is expressed on activated platelets. It has been reported that binding of HMGB1 to RAGE on neutrophils induces NET formation [44].

Fcγ receptors expressed on PMNs participate in the induction of ROS production as well as recognition of antibody-opsonized pathogens [46]. The immune complex increases NET formation in murine neutrophils expressing human FcγRIIa (CD32a), but not FcγRIIIb (CD16b) [47]; however, among the antibody receptors expressed by human neutrophils, only FcγRIIIb (CD16b) is responsible for NET formation in response to cross-linking antibodies [48]. Experimental differences, including cell types and stimulants, may be responsible for these differences in findings. Interestingly, TLR7/8 activation leads to proteolytic cleavage of FcγRIIa, thereby shifting neutrophils from phagocytosis of immune complexes to NET formation [49]. Clinically relevant evidence has revealed that neutrophils from patients with systemic lupus erythematosus (SLE) demonstrate similar cleavage of FcγRIIa related to that of neutrophil activation [49].

## 5. NETs and Cardiovascular Diseases

**Figure 2:**
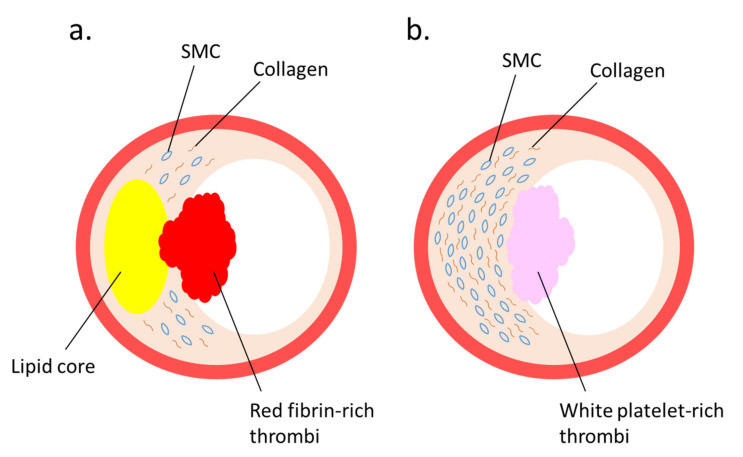
Two major lesions that lead to thrombus formation: (**a**) plaque rupture and (**b**) plaque erosion—are illustrated. (**a**) A vulnerable plaque is characterized by a large lipid core covered by a thin fibrous cap. When the fibrous cap is ruptured, red thrombus is produced. (**b**) A plaque erosion is characterized by a thickened intima enriched with smooth muscle cells (SMCs) and collagen fibers. When endothelial cells are injured, white-platelet-rich thrombus is produced.

Several studies regarding the pathophysiological roles of NETs have elucidated the implication of NETs [50] with coronary artery [51,52] and venous thrombosis [53]. Immunothrombosis and NET-induced thrombosis are important and widely studied phenomena [54,55,56]. Plaque rupture and plaque erosion are two major causes of atherothrombosis. Plaques prone to rupture are characterized by a large lipid core, thin fibrous cap, and numerous macrophages, but few smooth muscle cells (SMCs), which form red fibrin-rich thrombi. Plaque erosion, which causes white platelet-rich thrombi, is associated with enriched collagen, abundant SMCs, and little accumulation of lipids and foam cells [57] (Figure 2). Neutrophils infiltrate culprit lesions, including both ruptured and eroded plaques [58], via NET formation [59]. By activating neutrophils, circulating MPO levels are increased in patients with acute coronary syndrome [60]. Lipid-lowering pharmacotherapy with statins reduces plasma cholesterol levels and suppresses lipid accumulation in the lesions, thus exerting stabilizing effects on rupture-prone atherosclerotic plaques with reduced inflammation in the vascular tissue [57,61,62,63]; however, a remarkable number of patients have experienced cardiovascular events even after achieving a marked reduction in their cholesterol levels by statin therapy; accordingly, the underlying mechanism for superficial erosion has gained immense attention [64]. Presently, NETs are regarded as pivotal contributors in the formation of thrombi during superficial erosion [65,66]. This is in accordance with the observations that MPO levels in plasma and the density of MPO-positive cells in thrombi are elevated in acute coronary disease patients with eroded culprit plaques, but not in patients with ruptured culprit plaques [67,68,69].

NETs have been implicated in metabolic abnormalities such as diabetes. Neutrophils isolated from type 1 and type 2 diabetes are sensitive to NET formation, and the accelerated NETs impair wound healing [19]. Hyperglycemia is correlated with elevated white blood cells and reduced high-density lipoprotein (HDL) cholesterol levels. In diabetic mice, raising the levels of functional HDL, which accepts cholesterol from peripheral tissues, leads to regression of atherosclerosis, associated with decreased plaque inflammation and reduced NET formation in the plaque [70].

The mechanistic link between NET formation and atherosclerosis/thrombosis has been widely investigated. NETs evoke inflammatory responses of immune cells to upregulate inflammatory cytokines [71]. NETs and their components not only damage ECs directly [72,73], but also trigger activation of ECs to form a scaffold, leading to thrombosis. In pathophysiological conditions, including vascular diseases, ECs lose their endothelial phenotype and then acquire a mesenchymal phenotype, which is characterized by the expression of fibroblastic markers [74]. Endothelial-to-mesenchymal transition (EndMT) is involved in the progression of atherosclerosis [74,75,76]. In general, NETs are internalized into ECs through RAGE via clathrin-dependent endocytosis; however, when excess amounts of NETs remain on the extracellular surface, it induces EndMT of ECs to loosen cell–cell contact, in which NE acts as a proteolytic enzyme of VE-cadherin [77].

## 6. The Impact of Modified LDL and Its Components on Vascular Cells

Qualitative abnormality of LDL in circulation as well as an imbalance in LDL-cholesterol levels increase the risk of CVDs [78]. Lipoprotein components are susceptible to various modifications through enzymatic and non-enzymatic reactions. Among the lipoprotein components, lipids are the primary targets of oxidation reactions. OxLDL formation proceeds through preferential oxidation of lipid components containing a polyunsaturated fatty acid moiety. During phospholipid oxidation (PLs), the following three types of products are generated. First, unsaturated fatty acids at the *sn-2* position of PLs are oxidized to form hydroxy, hydroperoxy, or keto groups. Second, long-chain oxidized fatty acids at the *sn-2* position of PLs are cleaved to form short-chain products containing aldehyde or carboxylic acids. Third, the short-chain acyl group at the *sn-2* position of PLs is hydrolyzed to form lysophospholipids (lysoPLs) [79]. Lipoprotein-associated phospholipase A_2_ (Lp-PLA_2_), also known as platelet-activating factor-acetylhydrolase, plays a crucial role in producing large amounts of lysoPLs in LDL [80]. Spontaneous deacylation of oxidized PLs under physiological conditions is also a possible route for the production of lysoPLs [81]. Subsequently, lipid peroxidation products, including malondialdehyde and 4-hydroxynonenal, act on amino groups in protein components [82]. Notably, several LDL subfractions exert stronger pathogenic effects, which are generated by posttranslational modification or changes in the ratio of lipid to protein components. These newly generated products can act as oxidation-specific epitopes to trigger various inflammatory responses mediated by various scavenger receptors [6].

To date, several types of in vitro modified LDL, including copper-induced oxLDL, acetylated LDL, aggregated LDL, and enzymatically modified LDL (E-LDL), have been investigated to assess their effects on vascular cells [83]. E-LDL, prepared in vitro using trypsin and cholesterol esterase without oxidation of lipids, presents higher bioactivity than copper-induced oxLDL in foam cell formation of SMCs [84]. E-LDL was postulated to be produced by the presence of proteases in atherosclerotic lesions, such as urokinase-type plasminogen activator, matrix metalloproteinase-2 (MMP-2), and MMP-9 [85]; moreover, it has been immunohistochemically detected in human atherosclerotic lesions [85,86]. E-LDL upregulates the expression of IL-8 [87] and adhesion molecules such as intercellular adhesion molecule-1 (ICAM-1), platelet endothelial cell adhesion molecule-1, P-selectin, and E-selectin in ECs, which enhance the adhesion and transmigration of monocytes and T-lymphocytes [88]. E-LDL is incorporated in vascular SMCs by micropinocytosis and upregulates the expression of lectin-like oxLDL receptor 1 (LOX-1) to increase the uptake of oxLDL [89]. E-LDL also increases ICAM-1 expression in aortic SMCs [88]. Interestingly, E-LDL reveals potent cytotoxicity on PMNs; the free fatty acids present in E-LDL play a causative role in this toxicity. The toxicity of oxLDL is mimicked by linoleic acid, oleic acid, or arachidonic acid, and is abrogated by coincubation with albumin [90]. This study demonstrated that direct perturbation of the cell membrane of PMNs with fatty acids may cause cytotoxicity and lead to PMN death, predominantly by necrosis, as the apoptotic markers were not detected in the study. There is a possibility that NETs are involved in E-LDL-induced cell death of PMNs.

The production of oxidized lipids and subsequent protein adducts contributes to an increase in the electronegativity of lipoprotein particles. Electronegative LDL [LDL(−)] is a naturally occurring LDL subfraction that is enriched with negatively charged particles [91]. LDL(−) contains non-LDL apolipoproteins such as apoA-I and apoJ [92] as well as aggregated LDL with phospholipase C-like and sphingomyelinase activities [93,94]. LDL(−) has a higher content of diacylglycerol, ceramide, monoacylglycerol, and phosphorylcoline [93]. Moreover, LDL(−) is preferentially associated with Lp-PLA_2_ [95,96] and comprises more lysoPLs and nonesterified fatty acids than native LDL [97]. LDL(−) exerts highly proatherogenic effects by activating various cells [98] that are mediated by LOX-1 and PAF receptors. These two receptors mediate LDL(−)-induced endothelial apoptosis [99,100]. The binding of LDL(−) to ECs via LOX-1 mediates LDL(−)-induced premature vascular endothelial senescence [101]. Furthermore, LDL(−) also induces aggregation of platelets through the LOX-1 and PAF receptors, which promote platelet adhesion to ECs and thrombogenic state [102]. Interestingly, LDL(−) enhances the formation of triglyceride (TG)-rich lipid droplets in macrophages and releases more cytokines than copper-induced oxLDL [103].

Most recently, we reported the characterization of in vivo-oxLDL in the LDL(−) fraction released from atherosclerotic plaques of patients with acute myocardial infarction (AMI) [104]. In vivo oxLDL was modified with oxidized phosphatidylcholine (oxPC) and oxidized HDL [105]. While oxLDL, recognized using the anti-oxPC antibody, was detected in several fractions including LDL(−) fractions, oxLDL in LDL(−) was threefold higher in patients with AMI than in healthy subjects [104]. Our observation is in accordance with previous studies that reported an increase in oxLDL levels in plasma samples from patients with AMI. Proteins in LDL(−) contain apoB conjugated with oxPC. Such a modification could cause increased resistance to lysosomal hydrolysis in foam cells [106]. Moreover, LDL(−) is enriched with HDL-like particles containing apoA-I heavily modified with acrolein adducts. Electron microscopic analysis revealed that LDL(−) contains heterogeneous and aggregated subpopulations with small-sized particles, similar to HDL [104].

These results imply that small dense LDL (sdLDL) could be related to in vivo oxLDL, which has been reported as another atherogenic subfraction of LDL with high oxidative susceptibility [107] and a strong association with a future risk for coronary heart diseases [108,109]. Elevated levels of TG-rich VLDL in plasma cause increased synthesis of TG-rich LDL and subsequent extensive hydrolysis of core TG by hepatic lipase, which in turn leads to increased formation of sdLDL particles [110,111]. Indeed, elevated sdLDL levels are associated with increased postprandial large VLDL and remnant-like particles in patients with AMI [112]. The metabolic rate of sdLDL increases owing to its decreased affinity for LDL receptor, resulting in delayed hepatic clearance [113]. Furthermore, enhanced binding of sdLDL to extrahepatic tissues is mediated by increased binding to proteoglycans of the cell surface [114]. Foam cell formation in THP-1 macrophages, induced by stimulation with sdLDL, is associated with a remarkable increase in mRNA and protein expression of LOX-1 via TLR4 [107], thereby indicating that sdLDL may upregulate signal transduction through TLR4.

## 7. Modified LDL and NET Formation

**Figure 3:**
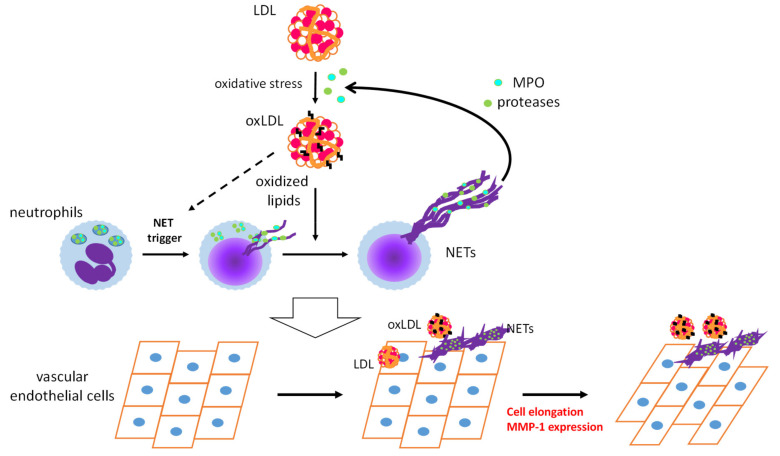
Schematic diagram of a feed-forward cascade that drives the neutrophil extracellular trap (NET)-induced endothelial inflammatory response. OxLDL strongly accelerates NET formation of activated neutrophils, while the same NET formation-inducing effect of oxLDL on resting neutrophils has not been observed. MPO and proteases released from neutrophils by NET formation could act on native low-density lipoprotein (LDL) to facilitate modification of LDL. Coexistence of NETs and oxLDL or native LDL promotes endothelial inflammation to cause cell elongation and enhanced MMP-1 production in endothelial cells [119]. MPO: myeloperoxidase, MMP-1: matrix metalloproteinase-1.

OxLDL acts as a DAMP and triggers sterile inflammatory responses to promote CVDs [6]. Infiltration of neutrophils into arteries during the early stages of atherosclerosis has been observed in hypercholesterolemic mice [115]. Numerous studies, on mice and humans, have provided evidence for the contribution of neutrophils to early atherosclerosis [116,117]. We have demonstrated the appearance of oxLDL in vascular tissues and in circulation even prior to atherosclerotic lesion development in apoE-KO mice [118]. These findings prompted us to examine whether oxLDL influences NET formation and subsequent endothelial inflammatory responses. PMA-induced NET formation in both HL-60-derived neutrophils and human PMNs is remarkably accelerated upon coincubation with oxLDL, but not with native LDL [119]. Moreover, extracellular components, after induction of NET formation by PMA with oxLDL, are capable of inducing morphological changes and MMP-1 expression in human aortic ECs (Figure 3). Presumably, neutrophils may play a pivotal role in the early stages of atherogenesis through the cooperative actions of oxLDL.

Intriguingly, NET-induced activation of human aortic ECs is enhanced upon coincubation with native LDL, suggesting that MPO and proteases released from neutrophils upon NET formation could act on native LDL and induce oxidative modification and/or degradation of LDL to produce modified proinflammatory LDL [11]. These phenomena, induced by the coexistence of NETs and native LDL or oxLDL, correspond to enhanced EndMT caused by the excess phagocytic capacity of ECs [77]. One possibility is that the cooperative action of oxLDL and NETs on ECs induces EndMT and subsequent neovascularization, which leads to atherosclerotic lesion formation [120].

**Figure 4:**
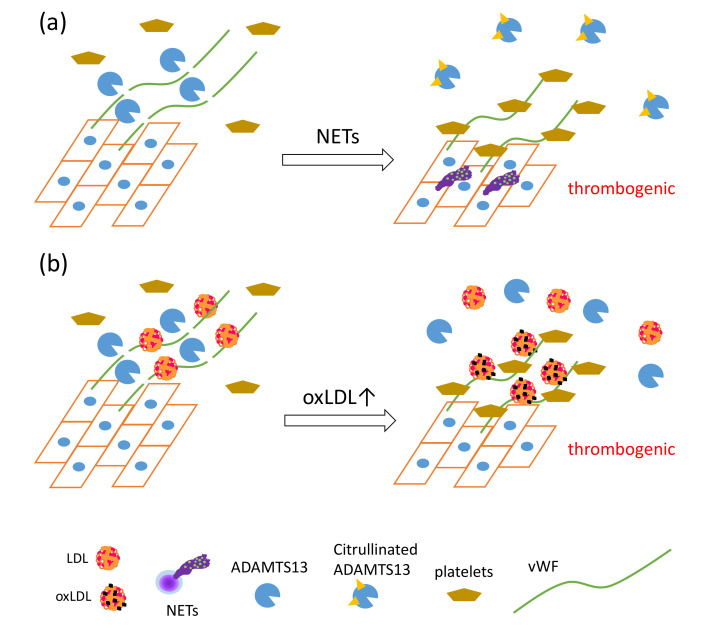
The synergistic actions of NET formation and oxLDL production presumably cause a proatherothrombotic status on the vascular endothelium. (**a**) PAD4 secreted by NET formation mediates citrullination of ADAMTS13, which inhibits the proteolytic activity of ADAMTS13 against von Willebrand factor (vWF), leading to increased vWF-platelet string formation on the endothelium [41]. (**b**) LDL, identified as a novel vWF-binding partner, accelerates the proteolytic cleavage of ultra-large vWF by ADAMTS13 under shear stress, and inhibits adhesion of platelets to ultra-large vWF. OxLDL, having lower ability to enhance proteolytic activity of ADAMTS13 than native LDL, competes with native LDL, which in turn increases platelet adhesion to ultra-large vWF, which could cause thrombus formation rich in vWF, platelets, and DNA [121].

Recently, a unique regulatory mechanism of lipoprotein-mediated vWF degradation has been described. LDL, but not HDL, has the ability to bind vWF and accelerate proteolytic cleavage of vWF by ADAMTS13 in a concentration-dependent manner under shear stress; however, oxLDL competitively inhibits vWF proteolysis associated with native LDL. Furthermore, native LDL, but not oxLDL, inhibits adhesion of platelets to vWF on ECs under flow [121]. NET formation also inhibits the proteolytic activity of ADAMTS13 via PAD4-mediated citrullination, as described earlier [41]. Considering that vWF functions as a scaffold for NETs, thrombosis, and platelets, these data indicate that oxLDL may enhance NET-mediated thrombosis formation via direct interaction with vWF (Figure 4).

Awasthi et al. reported the accelerating effect of oxLDL on NET formation in human neutrophils and proposed that components of oxLDL, such as oxPC and lysophosphatidylcholine, are responsible for NET formation [122]. Moreover, oxPLs derived from ether-linked phosphatidylethanolamine and phosphatidylcholine induce NET formation [123]. Furthermore, lipid extracts from LDL(−) of hypercholesterolemic patients induce calcium influx in human neutrophils, which is completely suppressed upon treatment with recombinant Lp-PLA_2_ [100], indicating that LDL(−) might contain bioactive oxPCs that affect NET formation. It has been reported that TLR2/6 acts synergistically on NET formation upon stimulation with oxLDL [122]. In addition, RAGE has been reported to mediate oxLDL-induced proinflammatory conditions in LDL receptor knockout mice [124]. RAGE-mediated neutrophil dysfunction [125] and HMGB1 expressed on platelets induce NET formation via RAGE [44]. These data suggest that oxLDL and oxidized lipids may initiate NET formation, and these products may act on neutrophils through TLRs and/or RAGE; however, the involvement of other scavenger receptors remains to be elucidated.

In terms of sphingolipids, an endogenous increase in ceramide has been implicated in the process of NET formation [126,127]. Interestingly, anacardic acid, a natural product extracted from cashew nut shells, stimulates neutrophils via sphingosine-1-phosphate (S1P) receptor 4 to induce NET formation [128]. Histone citrullination in mouse bone marrow neutrophils increases in a mouse model of fatty liver disease, in which the mice were fed a methionine-choline-deficient and high-fat diet, and a DNase injection attenuated the fatty liver of the mice [129]. This mouse fatty liver model suggested that hepatic S1P induces NET formation via S1P receptor 2, which plays a pivotal role in shifting the cell death of murine neutrophils from apoptosis to NETosis [129]. Considering that LDL(−) contains higher amounts of ceramides than normal LDL [93], and that HDL serves as a carrier of S1P [130], the role of lipoprotein sphingolipids would be of interest for future studies.

## 8. Significance of OxLDL in Autoimmune Diseases

SLE is an autoimmune disease associated with a high risk of CVDs, and neutrophils contribute to the pathogenesis of vascular damage via enhanced NET formation [131]. Low-density neutrophils (LDNs) are a unique subtype of neutrophils separated using gradient ultracentrifugation, which are increased in patients with various diseases, including autoimmune disease, cancer, and sepsis [132]. This subset of neutrophils is presumed to be in the activated state and serves as one of the key players in SLE [133]. LDNs from patients with SLE display distinct gene expression profiles in normal neutrophils, with higher expression of immunostimulatory and bactericidal proteins [134]. LDNs from patients with SLE exhibit higher susceptibility to NET formation and increased capacity to kill ECs through NET formation [134]. In fact, circulating oxLDL levels increase in patients with SLE, where oxLDL, but not native LDL, forms a complex with β2-glycoprotein I (β2-GPI). Such a complex could induce the production of proatherogenic anti-oxLDL/β2-GPI autoantibodies [135,136]. β2-GPI is a glycoprotein that specifically binds to oxidized products of phospholipids and cholesteryl esters and acts as a major target antigen for antiphospholipid antibodies developed in autoimmune diseases, including antiphospholipid syndrome and SLE [137]. Interestingly, the anti-β2-GPI/β2-GPI immune complex acts on neutrophils to induce NET formation associated with platelet aggregation, leading to enhanced thrombus formation [138]. These data indicate that the oxLDL/β2-GPI complex and/or its immune complex may also contribute to the development of vascular complications in SLE through upregulation of NET formation. It is noteworthy that LDNs express higher levels of LOX-1 in patients with SLE compared with the normal subjects [139]. Thus, presumably, LOX-1 may play a pivotal role in eliciting NET formation by stimulating neutrophils with oxLDL.

## 9. Conclusions

NET formation is induced by various stimulants, including exogenous DAMPs, in addition to numerous proinflammatory cytokines. Multiple receptors recognizing DAMPs, such as TLRs and RAGE, mediate NET formation by activating intracellular signal transduction. OxLDL and oxidized lipids induce and enhance NET formation. Considering that oxLDL and oxidized lipids are produced in the circulation, it is likely that oxLDL and oxidized lipids behave like endogenous DAMPs to affect neutrophil activation. Enzymes released from neutrophils after NET formation could modify LDL and HDL, which facilitates further neutrophil activation. Vascular damage through endothelial activation by NET formation leads to the development of atherosclerosis and thrombosis. Although further analysis is necessary, the concept that the synergistic action of oxLDL and neutrophils is linked to accelerated NET formation may provide new insight into the molecular and mechanistic basis of CVDs and aid the development of therapeutic approaches.

## Abbreviations

NETs	neutrophil extracellular traps
DAMPs	damage-associated molecular patterns
oxLDL	oxidized low-density lipoprotein
CVD	cardiovascular disease
MPO	myeloperoxidase
ROS	reactive oxygen species
NE	neutrophil elastase
PMNs	polymorphonuclear leukocytes
PMA	phorbol myristate acetate
PAD4	peptidylarginine deiminase 4
MS	mass spectrometry
PG	phenylglyoxal
ADAMTS13	a disintegrin-like and metalloproteinase with thrombospondin type 1 motif 13
vWF	von Willebrand factor
ECs	endothelial cells
TLRs	toll-like receptors
RAGE	receptor for advanced glycation end products
HMGB1	high-mobility group box 1
SLE	systemic lupus erythematosus
SMCs	smooth muscle cells
HDL	high-density lipoprotein
EndMT	endothelial-to-mesenchymal transition
PLs	phospholipids
Lp-PLA_2_	lipoprotein-associated phospholipase A_2_
E-LDL	enzymatically-modified LDL
LDL(-)	electronegative LDL
MMP	matrix metalloproteinase
LOX-1	lectin-like oxLDL receptor 1
ICAM-1	intercellular adhesion molecule-1
TGs	triglycerides
AMI	acute myocardial infarction
oxPC	oxidized phosphatidylcholine
sdLDL	small dense LDL
S1P	sphingosine-1-phosphate
LDN	low-density neutrophil
GPI	glycoprotein I
